# Bibliometric analysis of key biomarkers in sepsis-induced coagulopathy (2006–2025)

**DOI:** 10.1097/MD.0000000000051327

**Published:** 2026-10-30

**Authors:** Yidan Zhao, Huaxing Yao, Dong Lai, Pingping Xiao, Amei Li

**Affiliations:** a Department of Critical Care Medicine I, The Second Affiliated Hospital of Xiamen Medical College, Xiamen, Fujian, China; b Department of Pharmacy, The Second Affiliated Hospital of Xiamen Medical College, Xiamen, Fujian, China; c Department of Blood Transfusion, The Second Affiliated Hospital of Xiamen Medical College, Xiamen, Fujian, China; d Department of Hematology, The Second Affiliated Hospital of Xiamen Medical College, Xiamen, Fujian, China.

**Keywords:** bibliometrics, critically ill patients, research hotspots, sepsis-induced coagulopathy, SIC anticoagulant therapy, thrombin-antithrombin complex, thrombomodulin

## Abstract

Sepsis-induced coagulopathy is life-threatening in critically ill patients. Thrombin-antithrombin complex and thrombomodulin are key biomarkers, but no 20-year bibliometric analysis exists. We searched PubMed and Web of Science Core Collection (2006–2025) and analyzed 577 articles using CiteSpace and VOSviewer. Publications peaked in 2016 to 2018 and then declined. Japan, the United States, and China were the most productive; the United States had the highest citations. Hotspots focused on sepsis-induced coagulopathy, endothelial injury, and intensive care unit anticoagulation. Thrombin-antithrombin complex/thrombomodulin research is globally established, led by the United States. Future work should enhance international collaboration and develop multi-biomarker models.

## 1. Introduction

Sepsis-induced coagulopathy (SIC) is a common and life-threatening complication in critically ill patients. It is characterized by activation of coagulation, inhibition of fibrinolysis, and endothelial injury. In 2024, the updated Chinese expert consensus on the diagnosis and treatment of SIC further optimized diagnostic criteria and the risk stratification system.^[1]^ However, the roles of thrombin-antithrombin complex (TAT) and thrombomodulin (TM) as key diagnostic biomarkers in this updated framework have not yet been systematically summarized.^[2]^ Early and accurate diagnosis, along with timely intervention, is critical for improving patient prognosis. Thus, exploring specific and sensitive biomarkers remains central to research efforts in critical care medicine for SIC diagnosis. TAT, as a direct marker of thrombin activation, reflects the degree of coagulation activation. TM, meanwhile, serves as a specific indicator of endothelial cell injury. Both markers have been incorporated into SIC diagnostic systems and have emerged as key research topics.^[3,4]^

Studies investigating TAT and TM for SIC diagnosis have increased rapidly in recent years. However, comprehensive bibliometric analyses specifically examining TAT and TM as dual biomarkers in SIC remain lacking. Bibliometrics, an analytical approach based on statistical and visualization methods, enables objective and comprehensive exploration of publication patterns, academic structures, and research hotspots in a specific field. It also provides directional guidance for future research.^[3]^ To address this gap, we conducted the first systematic bibliometric analysis, distinct from traditional narrative reviews, focusing specifically on TAT and TM as dual biomarkers in SIC diagnosis from 2006 to 2025. Although previous reviews have summarized the roles of coagulation markers in sepsis, no prior study has provided a 20-year bibliometric overview of publication trends, collaboration networks, and evolving research hotspots specifically for TAT and TM in SIC. This study aimed to: analyze global publication trends; identify key countries, institutions, and authors; explore research hotspots and keyword bursts; and track topical changes to predict future research directions, thereby providing evidence-based references for basic research and clinical practice.

## 2. Materials and methods

### 2.1. Literature retrieval strategy

Data sources: PubMed and Web of Science Core Collection (WoSCC) databases were selected for their high accuracy in document-type annotation and comprehensive coverage of core global medical research. Limitations associated with database selection are addressed in Section 4.4.

Retrieval period: January 1, 2006, to December 31, 2025.

Language: English.

Retrieval strategies:

WoSCC: TS=(“antithrombin III-protease complex*” OR “thrombin-antithrombin III complex” OR “TAT*” OR “AT III-protease complex” OR “TM”) AND TS=(“Sepsis” OR “Septic coagulopathy” OR “Bloodstream Infection” OR “Infection, Bloodstream” OR “Septicemia” OR “Blood Poisoning” OR “Poisoning, Blood” OR “Severe Sepsis” OR “Septic Shock” OR “Pyemia”)

PubMed: (((“antithrombin III-protease complex”) OR (“thrombin-antithrombin III complex”) OR (“TAT”) OR (“AT III-protease complex”) OR (“TM”)) AND ((“Sepsis”) OR (“Bloodstream Infection”) OR (“Bloodstream Infections”) OR (“Infection, Bloodstream”) OR (“Septicemia”) OR (“Septicemias”) OR (“Blood Poisoning”) OR (“Blood Poisonings”) OR (“Poisonings, Blood”) OR (“Poisoning, Blood”) OR (“Severe Sepsis”) OR (“Sepsis, Severe”) OR (“Pyemia”) OR (“Pyemias”) OR (“Pyaemia”) OR (“Pyohemia”) OR (“Pyohemias”))).

### 2.2. Inclusion and exclusion criteria

Inclusion criteria: full-text studies related to TAT, TM, and SIC; original articles and systematic reviews published in English; articles published between 2006 and 2025; original research or systematic reviews (editorials, letters, and commentaries were excluded).

Exclusion criteria: literature not relevant to TAT, TM, or SIC; non-research document types, such as conference abstracts, news items, and briefings; literature available only in non-text form; editorials, letters, commentaries, and other non-original research articles.

### 2.3. Literature screening and data extraction

This bibliometric analysis follows the PRISMA-ScR guidelines for reporting.

Two independent researchers screened retrieved records based on titles, abstracts, and full texts, applying predefined inclusion and exclusion criteria. Any disagreements between the 2 researchers were resolved by discussion or adjudication by a senior third researcher. Extracted data were imported into an Excel database for subsequent analysis.

The detailed screening process is illustrated in Figure 1.

**Figure 1.**
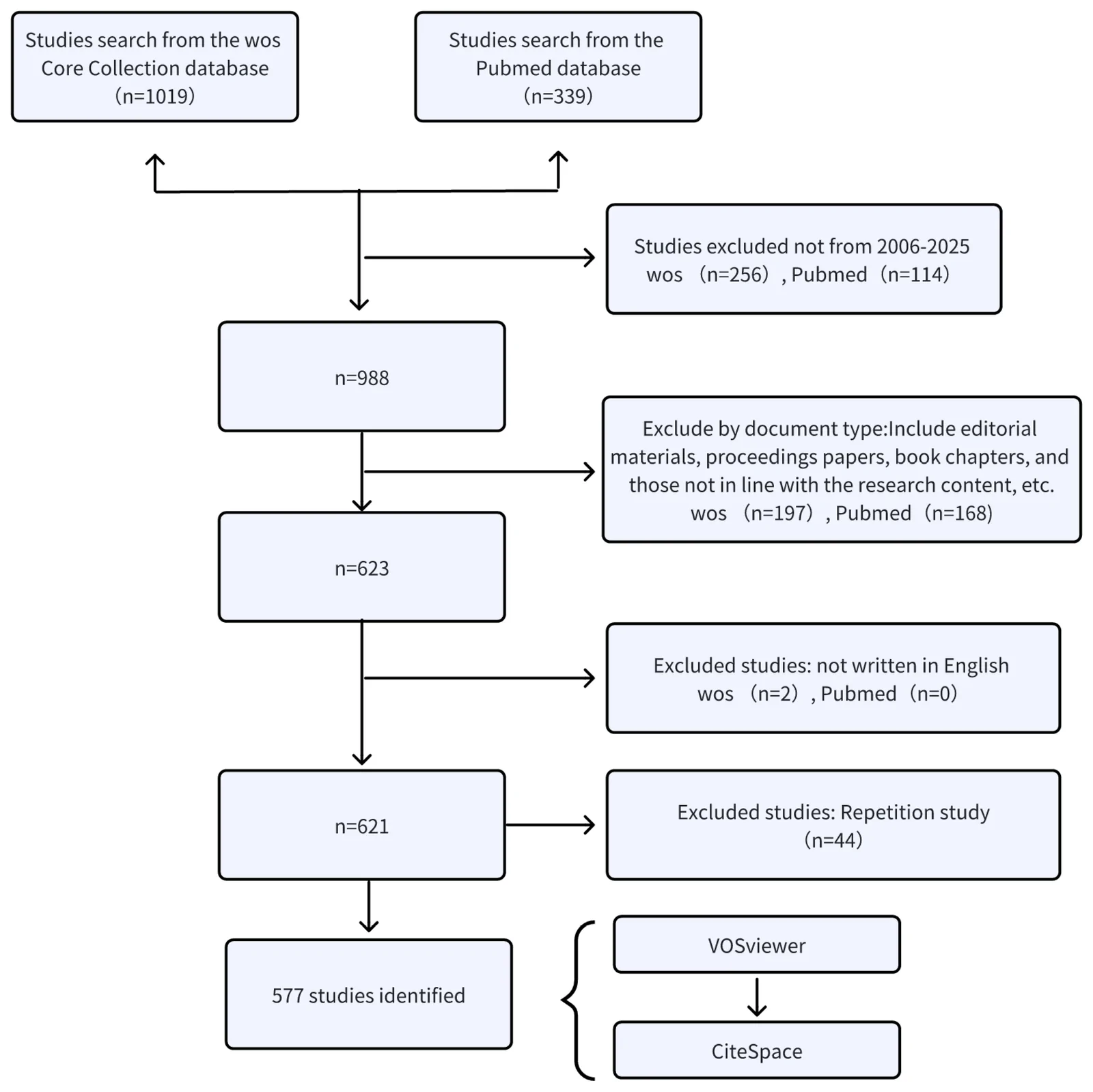
Flowchart of literature search and screening.

### 2.4. Data analysis methods

VOSviewer version 1.6.18 (Centre for Science and Technology Studies [CWTS], Leiden University) and CiteSpace version 6.2.4R (Drexel University) were employed for bibliometric analysis and visualization.

VOSviewer 1.6.18 was utilized to analyze collaboration patterns among countries, institutions, and authors, keyword co-occurrence, and journal publication density. A minimum occurrence threshold of 5 was applied to ensure the robustness of results.

CiteSpace 6.2.4R was used to assess annual publication trends, co-cited literature clusters, keyword burst detection, and timeline distribution.^[5]^ Parameters were set as follows: time slice = 1 year, pruning method = Pathfinder, and the top 50 items per slice were selected for analysis.

Primary indicators analyzed included annual publication volume, citation frequencies by country, institution, and author, journal impact factor (IF), keyword co-occurrence frequency, and keyword burst intensity and duration.

The raw data supporting the findings are available in the Table S1, Supplemental Digital Content 1.

## 3. Results

### 3.1. Literature retrieval results and annual publication trend

After strict screening of records from PubMed and WoSCC, a total of 577 articles published between January 1, 2006, and December 31, 2025, were finally included. Analysis of publication trends revealed a phased evolution within this research field. Between 2006 and 2015, the volume of published literature demonstrated a steady increase, primarily attributed to advancements in clinical definitions for sepsis (e.g., the formulation and adoption of Sepsis 3.0 criteria) and the widespread application of TAT and TM.^[2,6]^ These developments provided a solid foundation for subsequent basic and clinical research. A publication peak occurred between 2016 and 2018, mainly driven by the release of the 2016 International Consensus on Sepsis-3.0 and the first Diagnostic Criteria for SIC in 2017.^[4]^ The inclusion of TAT in these diagnostic guidelines prompted extensive confirmatory research globally. Since 2019, the number of publications has steadily declined, indicating that the research field has entered a bottleneck stage characterized by a need for deeper integration and innovative approaches. This stagnation largely results from the absence of personalized diagnostic strategies for SIC among critically ill patients with varying disease severities. Key contributing factors include: increasingly evident limitations of TAT and TM when used individually, particularly insufficient diagnostic specificity and real-time performance^[7,8]^ and redirection of global research resources toward Coronavirus Disease 2019 (COVID-19)-related coagulation disorders following the onset of the COVID-19 pandemic in 2020, which hindered progress in TAT/TM-based SIC diagnosis. In summary, research on TAT and TM in SIC diagnosis began with technological advancements and updates in conceptual understanding, reached a peak of validation triggered by international consensus, and subsequently entered a period of consolidation and exploration of new research directions, influenced by technical constraints, shifting research priorities, and major public health events.

As shown in Figure 2, the annual publication volume in this field increased rapidly from 2012 to 2018, peaking at 47 articles per year, and then gradually declined after 2019. The declining trend suggests that the field may stabilize at a lower publication volume by 2030, reflecting a mature research landscape.

**Figure 2.**
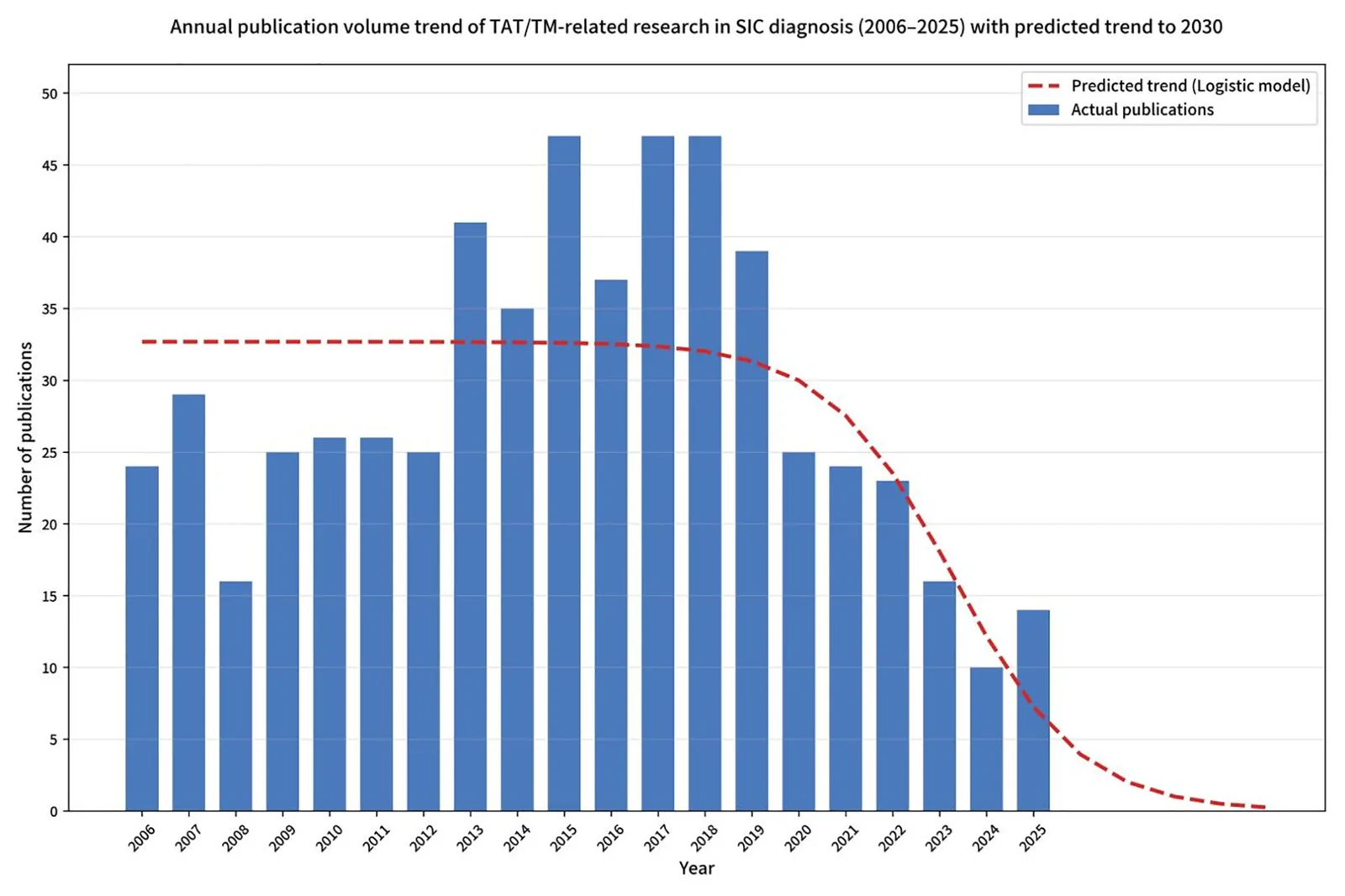
Annual publication volume trend for TAT/TM-related research in SIC diagnosis (2006–2025), with logistic growth projections extending to 2030 (dashed red line). SIC = sepsis-induced coagulopathy, TAT = thrombin-antithrombin complex, TM = thrombomodulin.

### 3.2. Analysis of research countries and regions

Table 1 and Figure 3 present the top 10 countries/regions with the highest publication counts over the past 2 decades. The leading 5 countries/regions in this field were Japan, the United States, China, the Netherlands, and the United Kingdom.

**Table 1:** Number of publications, total citations, and average citations in TAT/TM-related SIC diagnosis research by the top 10 countries/regions.

Rank	Country/region	Article counts	Percentage (%)	Total citations	Average citations
1	Japan	207	35.88%	7729	37.34
2	United States	160	27.73%	11286	70.54
3	China	67	11.61%	2852	42.57
4	Netherlands	61	10.57%	3336	54.69
5	England	43	7.45%	3322	77.26
6	Germany	27	4.68%	1208	44.74
7	France	26	4.51%	1346	51.77
8	Canada	23	3.99%	1173	51
9	Italy	22	3.81%	1210	55
10	Denmark	16	2.77%	751	46.94

Percentages reflect each country’s proportion of the total included studies (n = 577). The sum of percentages exceeds 100% due to rounding and additional contributions from other countries/regions.

SIC = sepsis-induced coagulopathy, TAT = thrombin-antithrombin complex, TM = thrombomodulin.

**Figure 3.**
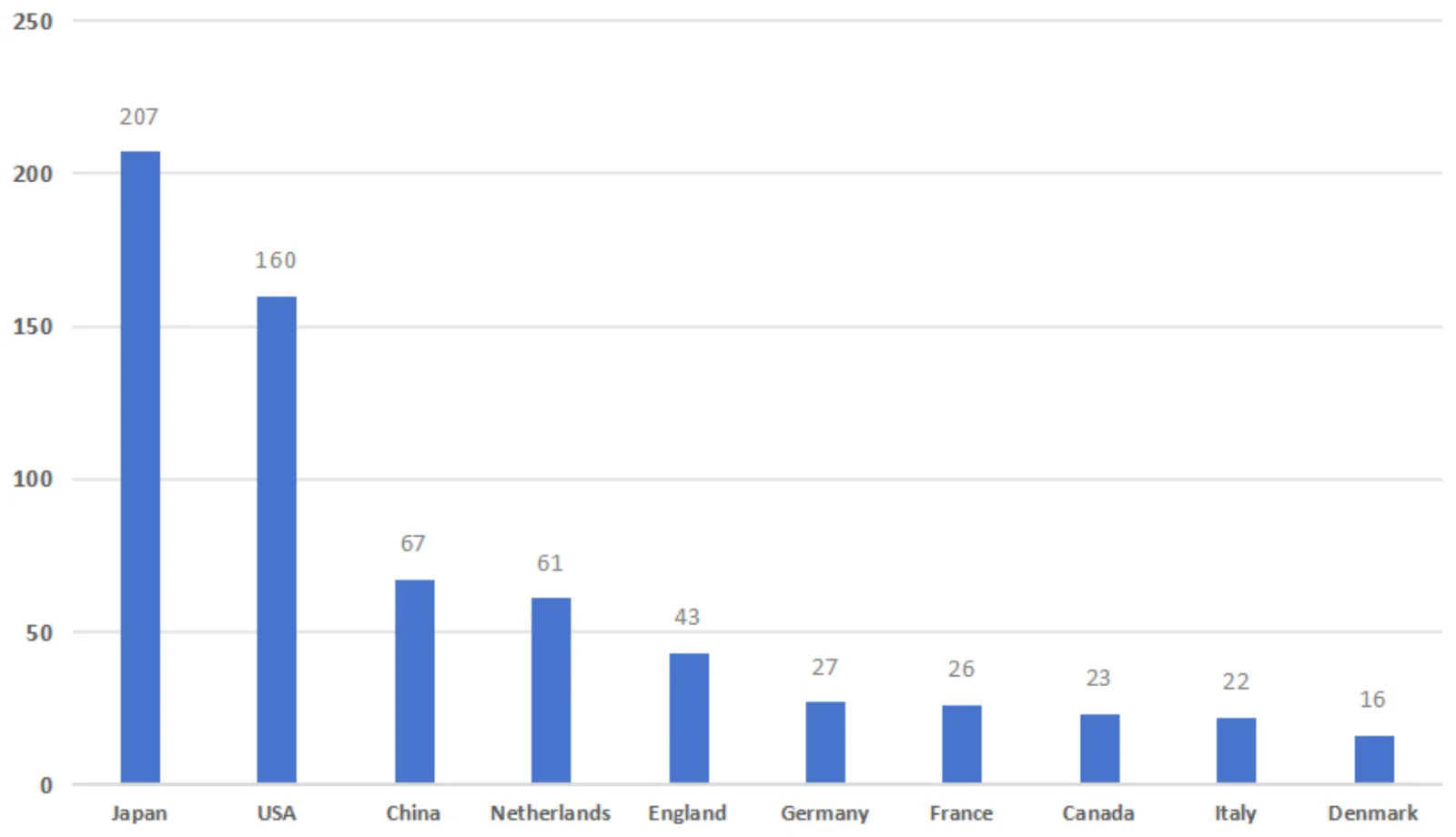
Publication volume on TAT/TM-related SIC diagnosis by the top 10 countries/regions. SIC = sepsis-induced coagulopathy, TAT = thrombin-antithrombin complex, TM = thrombomodulin.

Among these, the United States ranked first in both total publications and overall citations, with an average citation rate of 70.54 per article, clearly indicating its prominent role, particularly in coagulation-inflammation mechanisms associated with sepsis.^[9]^ Although Japan produced the highest number of publications (207 articles), its average citation frequency (37.34) was comparatively lower, suggesting potential for greater international impact. Percentages in Table 1 were calculated relative to the total number of included studies (n = 577); the sum of percentages may deviate from 100% due to rounding.

Figure 4 illustrates the international collaboration network, with Japan and the United States as core nodes. The United States, despite having fewer publications than Japan, acts as the central hub for international cooperation, partnering with Italy, Spain, China, Japan, and the Netherlands. This suggests a positive correlation between collaborative intensity and research impact: the United States’s extensive international collaboration aligns with its higher average citation rate (70.54), whereas Japan’s relatively limited cross-border cooperation might contribute to its lower citation frequency (37.34).

**Figure 4.**
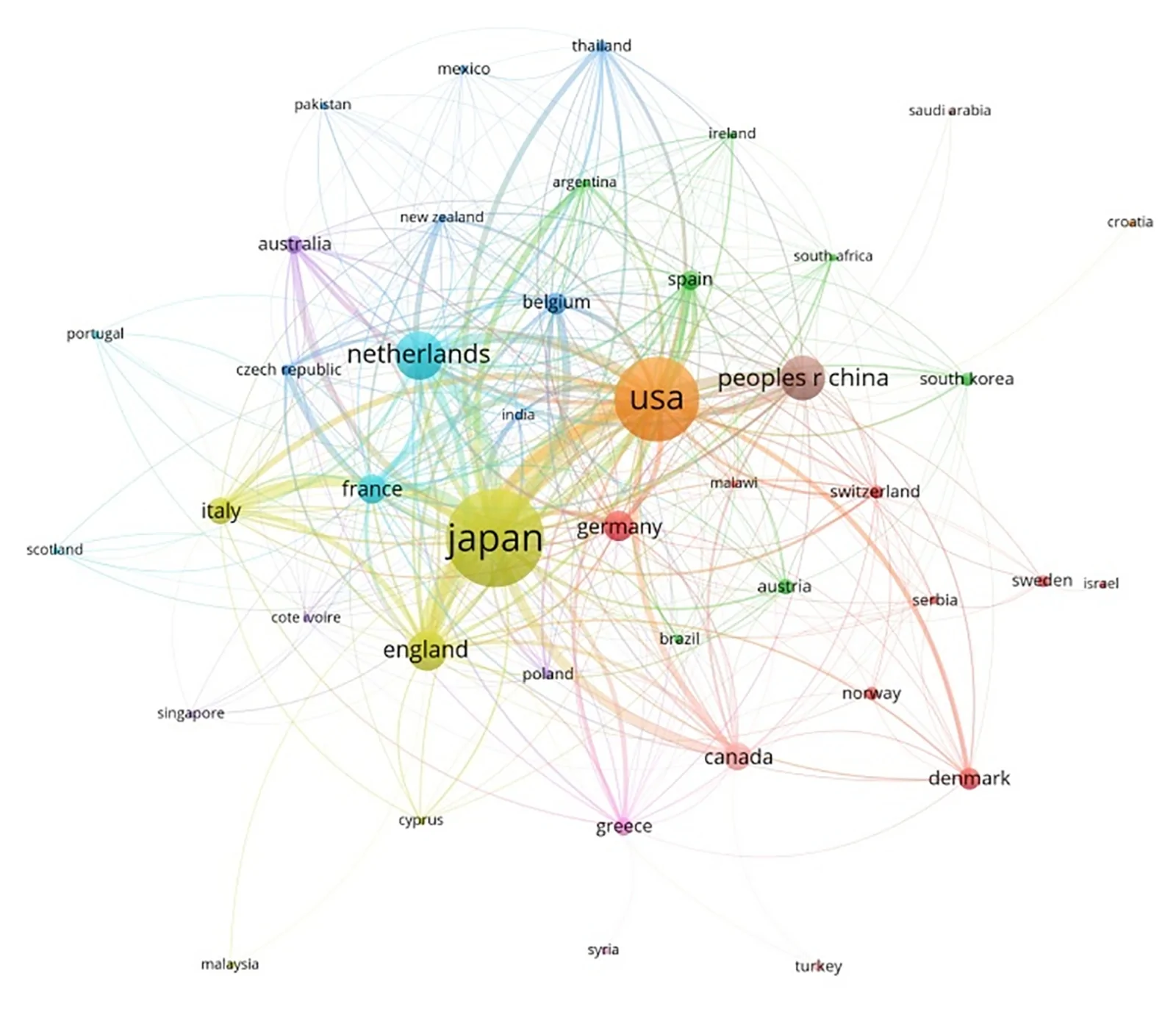
International collaborative network of TAT/TM research in SIC diagnosis (based on the PubMed database). SIC = sepsis-induced coagulopathy, TAT = thrombin-antithrombin complex, TM = thrombomodulin.

This study integrated literature data from PubMed and WoSCC to construct a comprehensive dataset. However, VOSviewer supports the generation of network maps from only a single standardized data source and cannot directly merge multi-database files. To ensure data consistency and software compatibility, the international cooperation network map in this study was created using data from the PubMed database, which is authoritative in the life sciences and medical fields, effectively reflecting core international cooperation patterns in this field.

Node size represents the publication count from each corresponding country, node color indicates regional clustering, and thicker lines reflect stronger collaborative ties between countries.

### 3.3. Analysis of research institutions

The 10 most productive institutions in this research area are detailed in Table 2 and depicted in Figure 5. The University of Amsterdam (Netherlands) ranked first with 46 publications (2472 total citations, 53.74 average citations), followed by Juntendo University (Japan, 40 papers, 2255 total citations, 56.38 average citations) and Mie University (Japan, 19 papers, 467 total citations, 24.58 average citations).

**Table 2:** Publication output, total citations, and average citations of the top 10 research institutions in TAT/TM-related SIC diagnosis.

Rank	Institution	Country	Documents (n)	Citations (n)	Average citations
1	Univ of Amsterdam	Netherlands	46	2472	53.74
2	Juntendo University	Japan	40	2255	56.38
3	Mie University	Japan	19	467	24.58
4	Osaka General Medical Center	Japan	16	932	58.25
5	Kagoshima University	Japan	16	1442	90.13
6	Duke University	United States	16	1433	89.56
7	Osaka University	Japan	15	1055	70.33
8	Oklahoma Medical Research Foundation	United States	15	1004	66.93
9	University of Pennsylvania	United States	13	378	29.08
10	Asahi Kasei Pharma Corporation	Japan	13	440	33.85

SIC = sepsis-induced coagulopathy, TAT = thrombin-antithrombin complex, TM = thrombomodulin.

**Figure 5.**
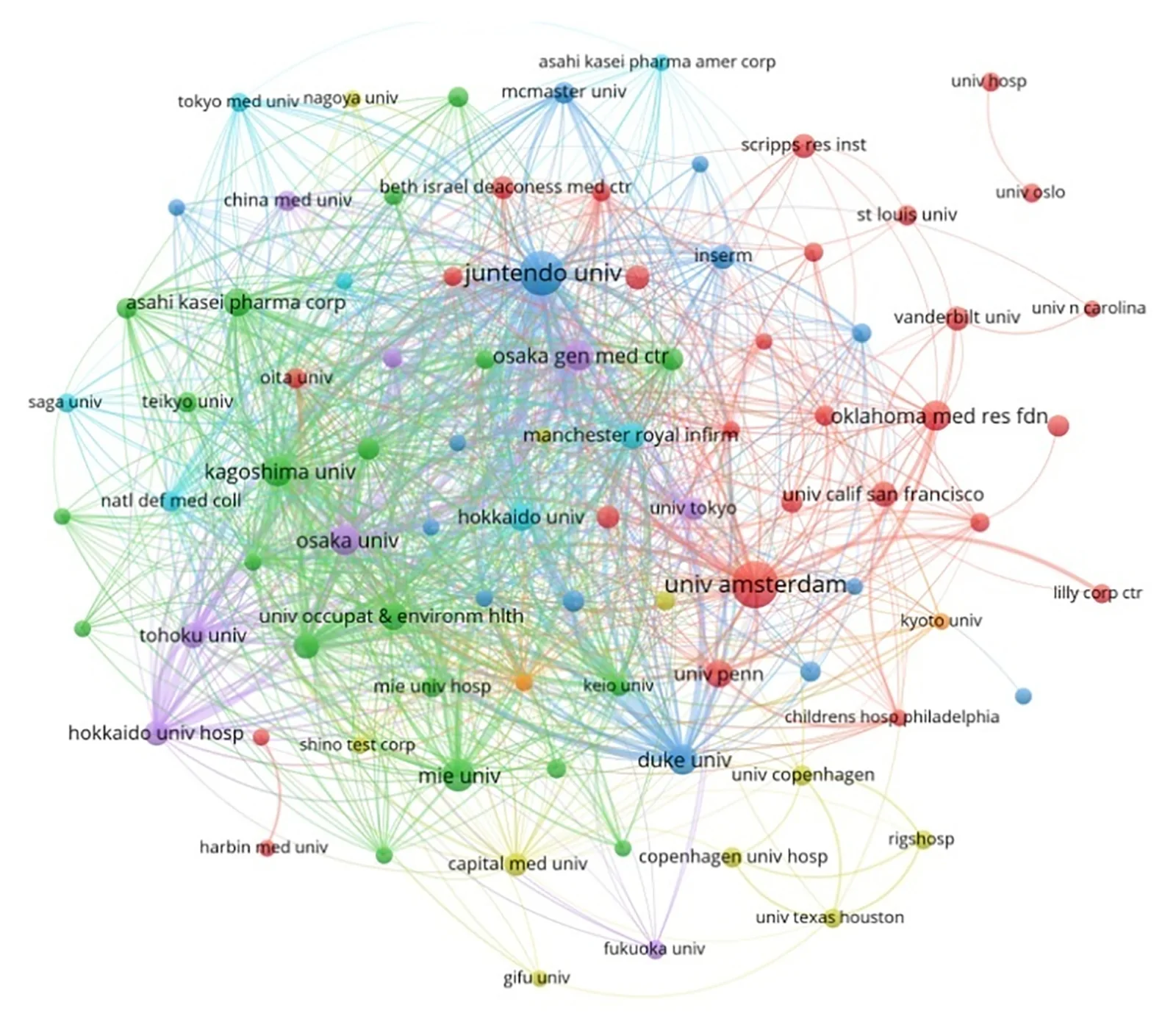
Collaborative network of core research institutions in TAT/TM-related SIC diagnosis. SIC = sepsis-induced coagulopathy, TAT = thrombin-antithrombin complex, TM = thrombomodulin.

Figure 5 demonstrates that core institutions tend to form regional clusters. Japanese institutions primarily established close domestic collaborative networks, whereas European and American institutions emphasized cross-border collaborations. Chinese institutions exhibited relatively weak connections to international core nodes, consistent with the previously noted lack of sufficient international cooperation.

Further analysis indicated that research institutions favored high-level domestic collaborations, while international and cross-regional cooperation remained limited.^[10]^ This may constitute a significant barrier to innovative advancement in this field. Therefore, enhancing global academic exchanges and institutional collaborations is essential to overcome geographical limitations, thus promoting cross-regional collaborative innovation and the broader dissemination of research findings.

Node size indicates the number of publications per institution, and node color denotes distinct institutional clusters.

### 3.4. Analysis of core authors

The top 10 authors ranked by both publication count and co-citation frequency are presented in Table 3. These 10 most productive authors contributed 178 articles, accounting for 30.85% of the entire literature corpus. Iba T (Japan) ranked first in publication volume (34 articles), followed by Van der Poll T (Netherlands, 24 articles) and Levi M (Netherlands, 23 articles). Regarding co-citation frequency, Levi M was cited most frequently (595 times), followed by Iba T (448 times) and Gando S (357 times).

**Table 3:** Publication productivity and co-citation frequency of the top 10 authors in TAT/TM-related SIC diagnosis research.

Rank	Author	Count	Rank	Co-cited author	Citation
1	Iba, T.	34	1	Levi, M.	595
2	Van der Poll, T.	24	2	Iba, T.	448
3	Levi, M.	23	3	Gando, S	357
4	Levy, J. H.	16	4	Vincent, J. L.	306
5	Yamakawa, K.	15	5	Esmon, C. T.	285
6	Wada, H.	15	6	Wada, H.	249
7	Esmon, C. T.	15	7	Taylor, F. B.	213
8	Thachil, J.	13	8	Yamakawa, K.	211
9	Roelofs, J. J. T. H.	12	9	Bernard, G. R.	185
10	Maruyama, I.	11	10	Abraham, E.	156

SIC = sepsis-induced coagulopathy, TAT = thrombin-antithrombin complex, TM = thrombomodulin.

Figure 6 illustrates the author collaboration network, highlighting 2 core research groups: the Dutch team, centered around Levi M and Van der Poll T, and the Japanese team, led by Iba T and Gando S. The formation of these stable research groups provides a solid foundation for ongoing and in-depth advancements in TAT and TM studies related to SIC diagnosis.

**Figure 6.**
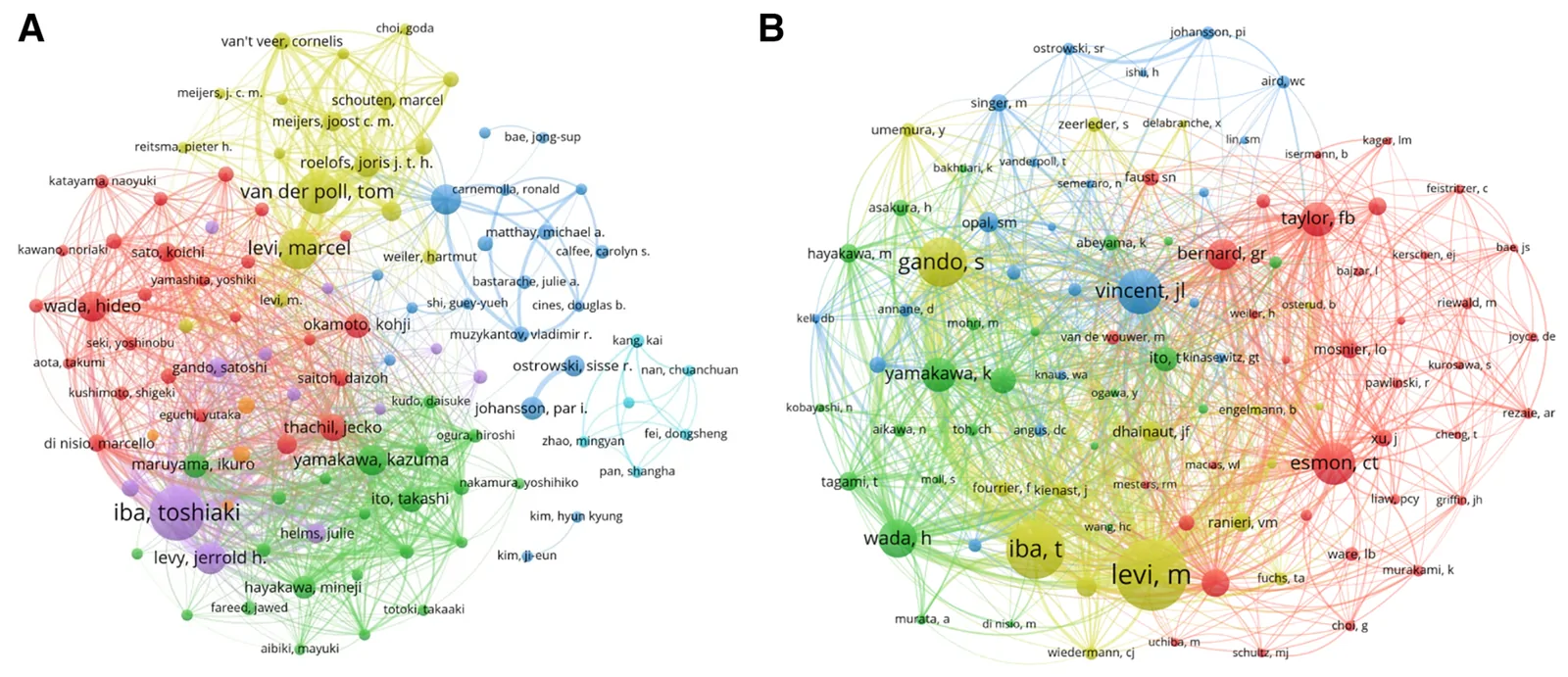
(A) Collaborative network of core authors. Node size represents publication volume per author, and node color indicates distinct author collaboration clusters. (B) Co-citation network of key authors. Node size reflects number of co-citations received, and node color distinguishes different co-citation clusters. Thicker lines denote stronger co-citation relationships. Collaborative and co-citation networks of core authors in TAT/TM research for SIC diagnosis. SIC = sepsis-induced coagulopathy, TAT = thrombin-antithrombin complex, TM = thrombomodulin.

Figure 6A highlights the research focus of these 2 author teams: the Dutch group emphasizes coagulation-inflammation mechanisms, whereas the Japanese group concentrates on clinical biomarker applications. Figure 6B illustrates academic connections among key authors, identifying Levi M as the central figure linking different research clusters.

### 3.5. Analysis of published journals

Table 4 lists the top 10 journals publishing research in this field, primarily focused on thrombosis, hemostasis, critical care, and sepsis. The frequent appearance of these journals in the top 2 quartiles underscores the rigorous publication standards of this research area. Blood, with an IF of 23.1, emerged as the most influential journal, highlighting its central position in coagulation and hematology research.

**Table 4:** Publication volume, IF, and quartile rankings of the top 10 journals publishing TAT/TM-related SIC diagnosis research.

Rank	Journal	Article counts	Percentage (577)	IF (2025)	JCR quartile
1	Thromb Res	35	6.07%	3.4	Q2
2	J Thromb Haemost	26	4.51%	5.0	Q1
3	Critical Care	25	4.33%	9.3	Q1
4	Shock	23	3.99%	2.9	Q1
5	Critical Care Medicine	21	3.64%	6.0	Q1
6	Clin Appl Thromb Hemost	20	3.47%	2.0	Q3
7	Thromb Haemost	17	2.95%	4.3	Q1
8	PLoS One	15	2.60%	2.9	Q1
9	Blood	11	1.91%	23.1	Q1
10	J Crit Care	11	1.91%	2.9	Q2

IF = impact factor, JCR = journal citation reports, SIC = sepsis-induced coagulopathy, TAT = thrombin-antithrombin complex, TM = thrombomodulin.

Table 5 ranks the top 10 journals by co-citation frequency. J Thromb Haemost (2282 co-citations) was ranked first, followed by Critical Care (1439) and Thromb Res (1393). JAMA, with the highest IF (55.0), signifies its considerable academic influence in the field.

**Table 5:** Co-citation frequency, IF, and quartile of the top 10 cited journals in TAT/TM-related SIC diagnosis research.

Rank	Cited journal	Co-citation	IF	Quartile in category
1	J Thromb Haemost	2282	5.0	Q1
2	Critical Care	1439	9.3	Q1
3	Thrombosis Research	1393	3.4	Q2
4	Blood	1251	23.1	Q1
5	Critical Care Medicine	1201	6.0	Q1
6	Shock	1060	2.9	Q1
7	JAMA	886	55.0	Q1
8	Int J Mol Sci	638	4.9	Q1
9	Thromb Haemos	572	4.3	Q1
10	Semin Thromb Hemost	547	4.1	Q2

IF = impact factor, JAMA = Journal of the American Medical Association, SIC = sepsis-induced coagulopathy, TAT = thrombin-antithrombin complex, TM = thrombomodulin.

Figure 7A confirms J Thromb Haemost, Critical Care, and Blood as core journals. Figure 7B illustrates how molecular biology journals frequently inform research published in clinical medicine journals, reflecting an integration between basic and clinical research domains.

**Figure 7.**
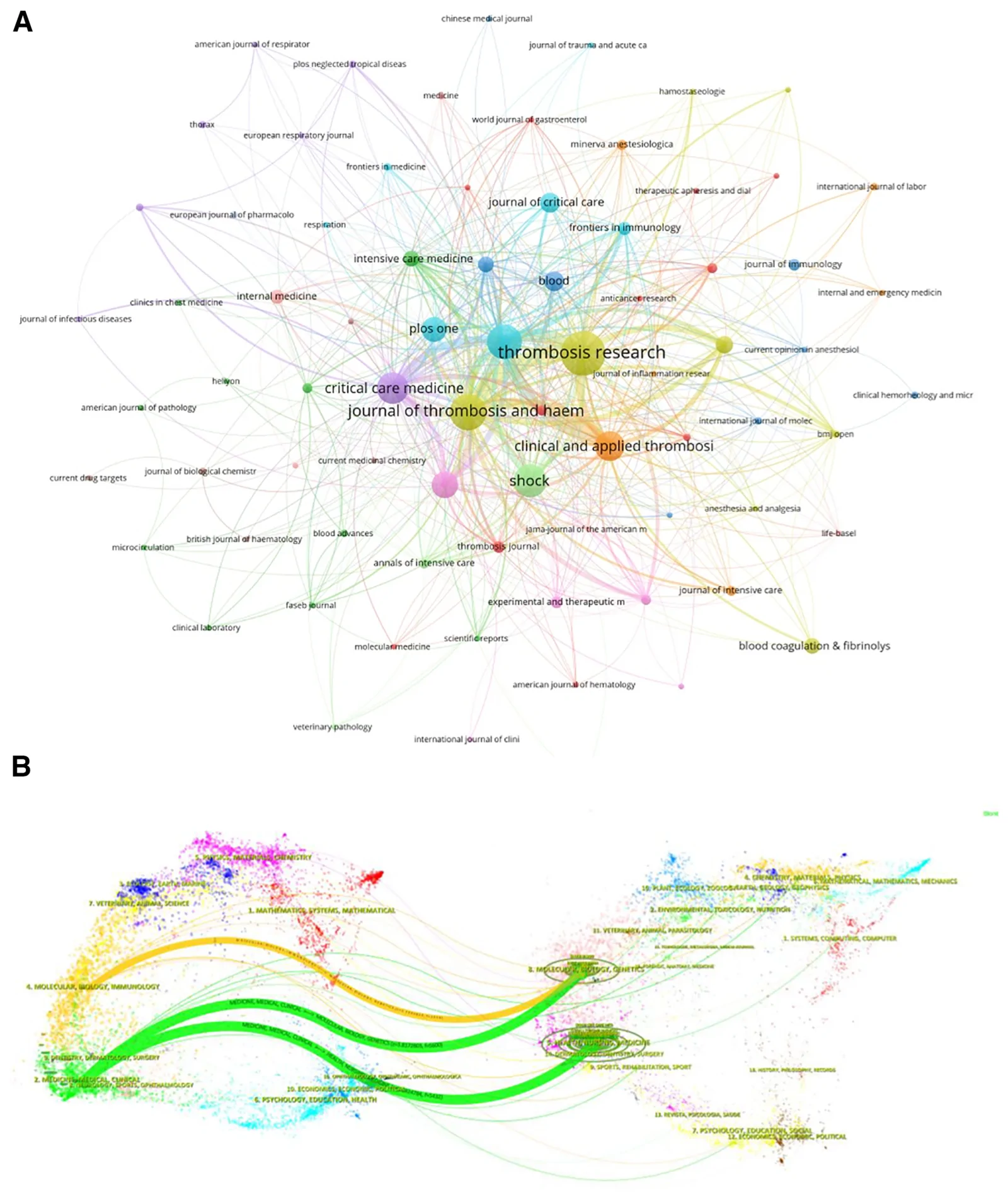
(A) Citation network of journals publishing TAT/TM-related SIC diagnosis research. Node size represents journal citation counts, and node color indicates citation-based journal clusters. (B) Dual-map visualization of citation patterns. Left side: citing journals; Right side: cited journals. Arcs indicate citation flows; thicker arcs represent stronger citation links. Green: molecular biology/immunology → clinical medicine; Yellow: molecular biology/immunology → genetics. SIC = sepsis-induced coagulopathy, TAT = thrombin-antithrombin complex, TM = thrombomodulin.

Node size represents journal citation counts, and node color indicates citation-based journal clusters.

The dual-map visualization (Fig. 7B) highlights thematic trends in published literature. Citation links, represented by colored arcs, connect citing journals (left) to cited journals (right). Two main citation paths emerged: molecular biology/immunology research frequently informs molecular biology/genetics and medicine/clinical studies.

### 3.6. Co-cited references

As presented in Table 6, the most frequently co-cited studies in this field concentrate on diagnostic criteria for SIC, anticoagulant therapy for SIC in intensive care units (ICUs), and the mechanistic roles of TAT and TM in coagulation. Figure 8 categorizes core co-cited references into 3 main clusters: diagnostic criteria, anticoagulant therapy, and biomarker mechanisms. Further, Figure 9 subdivides these clusters into 16 specific research themes, prominently featuring SIC and recombinant TM. Additionally, Figure 10 outlines the evolving research focus: initially on basic mechanisms (prior to 2010), subsequently on clinical trials (2010–2020), and currently highlighting endothelial cell injury (post-2020). These references form the foundational knowledge base of TAT/TM research in SIC diagnosis and significantly inform future studies. The most cited reference, a randomized controlled trial evaluating recombinant human activated protein C in severe sepsis, demonstrated the efficacy of anticoagulant therapy for improving prognosis, thereby pioneering SIC anticoagulation research. This trial provided robust clinical evidence confirming that recombinant human activated protein C reduces mortality in severe sepsis through anticoagulant, anti-inflammatory, and profibrinolytic effects.^[11]^ The second-most cited work, a phase III clinical trial assessing recombinant human soluble TM (ART-123) for disseminated intravascular coagulation (DIC), showed superior effectiveness and safety of ART-123 by targeting the coagulation-inflammation pathway.^[7]^ This finding validated TM’s clinical efficacy and tolerability in managing coagulation disorders, providing crucial support for its application in both the diagnosis and treatment of SIC.

**Table 6:** Top 10 highly co-cited literature in TAT/TM-related SIC diagnosis research.

Rank	Title	Journal	Total citations	DOI
1	RCT of recombinant human activated protein C for severe sepsis	N Engl J Med	144	10.1056/NEJMoa010330 (PMID: 11496836)
2	Phase III RCT of recombinant human soluble TM (ART-123) in disseminated intravascular coagulation	J Thromb Haemost	137	10.1111/j.1538-7836.2007.02564.x
3	Definition, clinical/laboratory criteria, and scoring system for disseminated intravascular coagulation	Thromb Haemost	108	10.1111/j.1538-7836.2009.03310.x
4	High-dose antithrombin III in severe sepsis: a randomized controlled trial	JAMA	103	10.1001/jama.283.10.1054
5	Phase 2b placebo-controlled RCT of recombinant human soluble TM (ART-123) in sepsis-related suspected disseminated intravascular coagulation	Crit Care Med	93	10.1097/01.CCM.0000245744.21774.8D
6	Multicenter prospective validation of disseminated intravascular coagulation diagnostic criteria in critically ill patients	Crit Care Med	90	10.1097/01.CCM.0000200334.66424.1E
7	Drotrecogin alfa (activated) for adult septic shock	N Engl J Med	85	10.1056/NEJMoa010254
8	Third International Consensus Definitions for Sepsis and Septic Shock (Sepsis-3)	JAMA	76	10.1001/jama.2016.0287
9	Antiinflammatory effect of TM N-terminal domain via high-mobility group-B1 sequestration	J Clin Investig	73	10.1172/JCI85652
10	High-dose antithrombin without heparin in severe sepsis with/without disseminated intravascular coagulation	J Thromb Haemost	72	10.1111/j.1538-7836.2005.01325.x

JAMA = Journal of the American Medical Association, RCT = randomised controlled trial, SIC = sepsis-induced coagulopathy, TAT = thrombin-antithrombin complex, TM = thrombomodulin.

**Figure 8.**
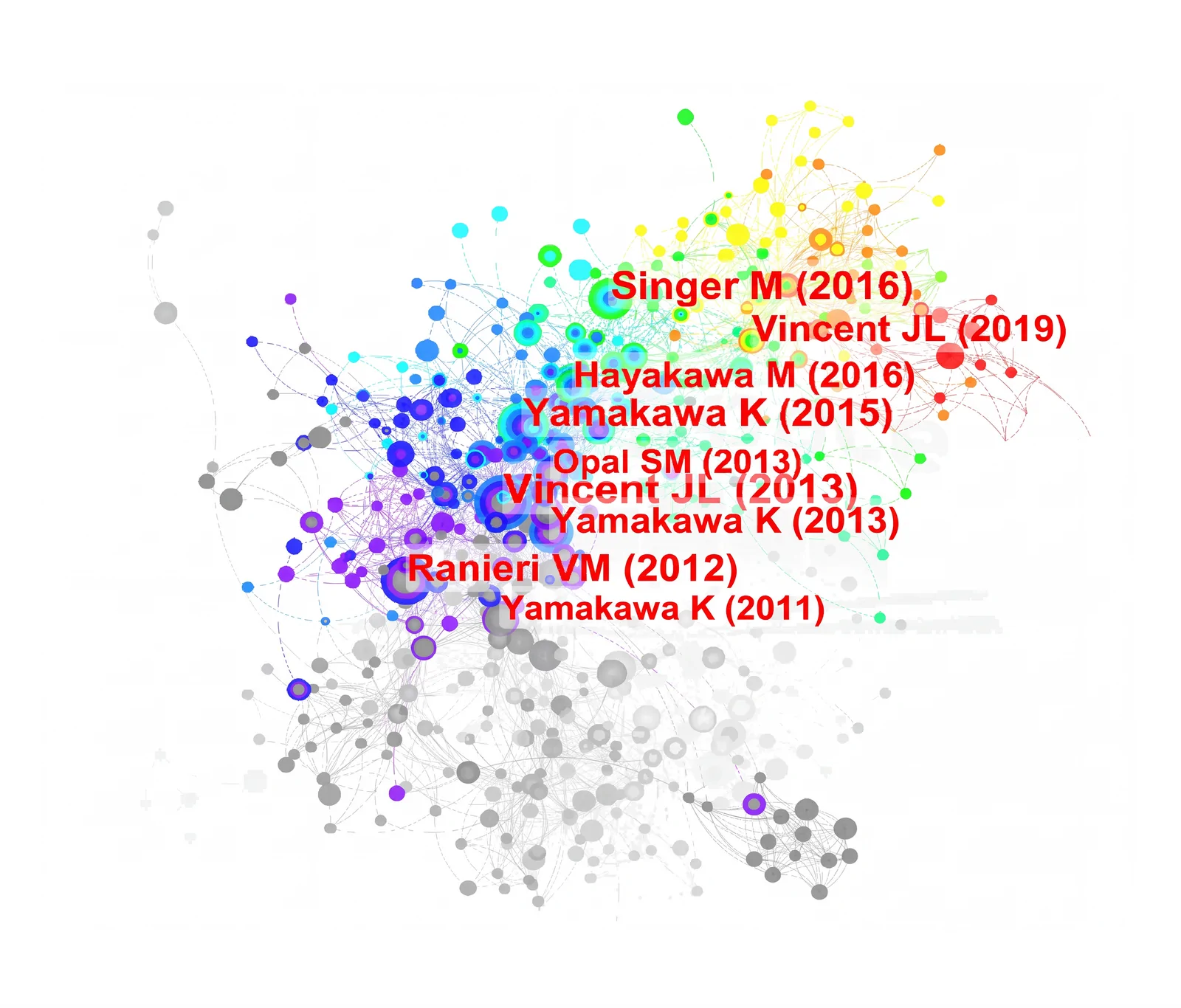
Co-citation network of core references on TAT/TM in SIC diagnosis. SIC = sepsis-induced coagulopathy, TAT = thrombin-antithrombin complex, TM = thrombomodulin.

**Figure 9.**
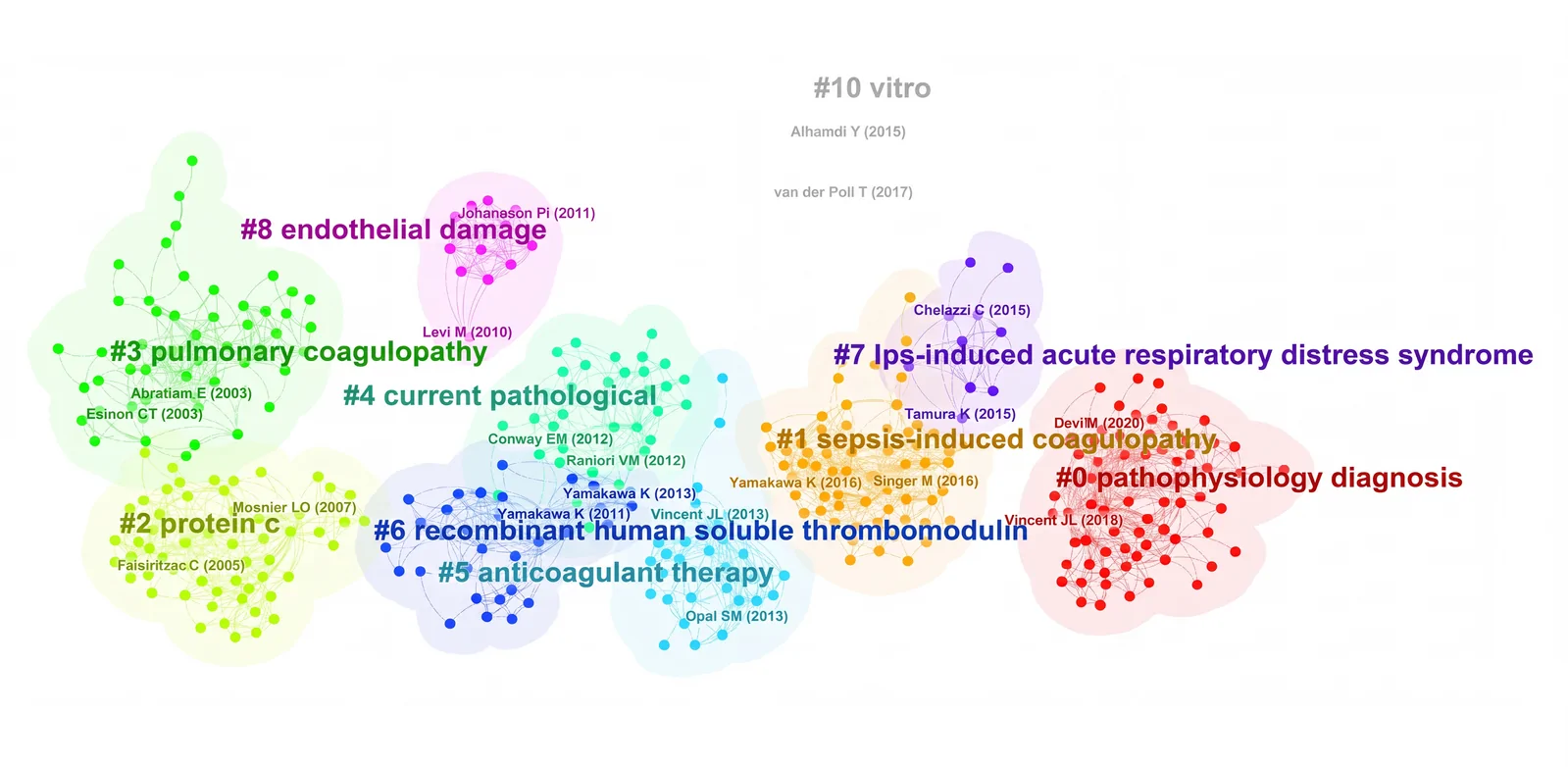
Cluster visualization of co-cited references in TAT/TM-related SIC diagnosis research. SIC = sepsis-induced coagulopathy, TAT = thrombin-antithrombin complex, TM = thrombomodulin.

**Figure 10.**
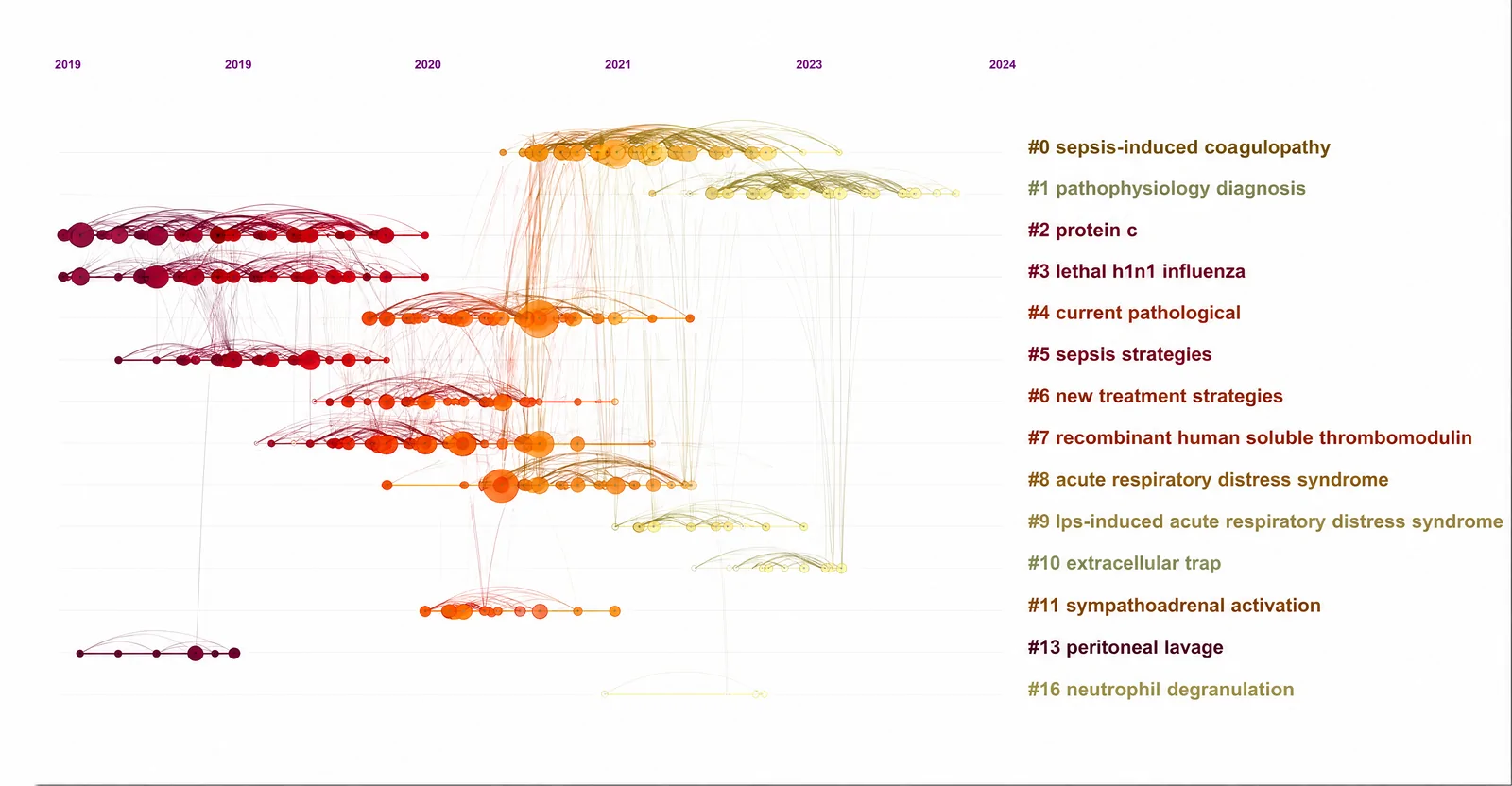
Timeline diagram illustrating the evolution of research hotspots in TAT/TM-related SIC diagnosis. SIC = sepsis-induced coagulopathy, TAT = thrombin-antithrombin complex, TM = thrombomodulin.

### 3.7. Research hotspots and evolutionary trends

Co-citation clustering analysis (Fig. 9) highlighted clearly defined research subdivisions. The core thematic clusters included SIC and recombinant human soluble TM, while secondary clusters encompassed pathophysiological diagnosis and acute respiratory dysfunction. The evolution of research hotspots (Fig. 10) demonstrated a progressive shift from early-stage fundamental mechanisms (e.g., protein C pathway, pathophysiology) to later-stage clinical applications (e.g., recombinant TM, sepsis treatment strategies), reflecting the transition from foundational to translational research.

Notably, these core clusters closely align with the top 10 highly co-cited references (Table 6), which include landmark clinical trials involving recombinant human activated protein C and soluble TM, as well as consensus definitions for sepsis and DIC. These impactful studies have substantially shaped core research themes and guided subsequent research directions, underscoring the crucial role of clinical evidence in advancing TAT/TM-based SIC diagnostics.

Node size represents co-citation frequency, reflecting academic influence. Node color differentiates thematic clusters, labeled by their core research topics (e.g., #0 SIC, #7 recombinant human soluble TM). Thicker lines indicate stronger co-citation relationships among references.

Each horizontal line represents a thematic cluster (labeled on the right), with node size corresponding to the cluster’s frequency per year. The color gradient (from red to yellow) reflects publication years within clusters, visually demonstrating temporal shifts and persistence of research topics.

### 3.8. Keyword analysis

Table 7 lists the top 20 high-frequency keywords. Keyword co-occurrence analysis (VOSviewer) identified the most relevant and core keywords for TAT/TM-based SIC diagnosis research, after excluding overly broad terms such as “sepsis”: specifically, “TM” (192), “disseminated intravascular coagulation” (239), “TAT,” and “severe sepsis” (169; Table 7, Figs. 11 and 12).^[3,12]^ Filtering non-substantive or general keywords resulted in a network of 88 high-frequency terms (occurrence ≥ 13), divided into 4 distinct clusters: pink (43 keywords), red (29), blue (10), and yellow (6). Research hotspots and their evolution were visualized using a keyword clustering map generated by CiteSpace (Fig. 13). Figure 11 illustrates that “TM” and “disseminated intravascular coagulation (DIC)” constitute the core keywords, while “severe sepsis” provides a clinical context rather than a central research theme. Figure 12 emphasizes that coagulation and inflammation represent concentrated research areas, particularly highlighting TAT and TM as diagnostic biomarkers. Figure 13 identifies 4 principal research directions: SIC mechanisms, TAT/TM biomarker applications, coagulation-inflammation interactions, and anticoagulant therapy, all firmly centered on TAT/TM-associated SIC diagnosis.

**Table 7:** Top 20 high-frequency keywords in TAT/TM-related SIC diagnosis research (2006–2025).

Rank	Keyword	Counts	Rank	Keyword	Counts
1	sepsis	298	11	antithrombin	70
2	disseminated intravascular coagulation	239	12	efficacy	68
3	TM	192	13	thrombosis	66
4	severe sepsis	169	14	safety	63
5	coagulation	138	15	critically ill patients	62
6	inflammation	127	16	activation	57
7	human soluble TM	125	17	double-blind	57
8	septic shock	112	18	multicenter	53
9	mortality	86	19	tissue factor	53
10	activated protein C	72	20	protein-c	50

SIC = sepsis-induced coagulopathy, TAT = thrombin-antithrombin complex, TM = thrombomodulin.

**Figure 11.**
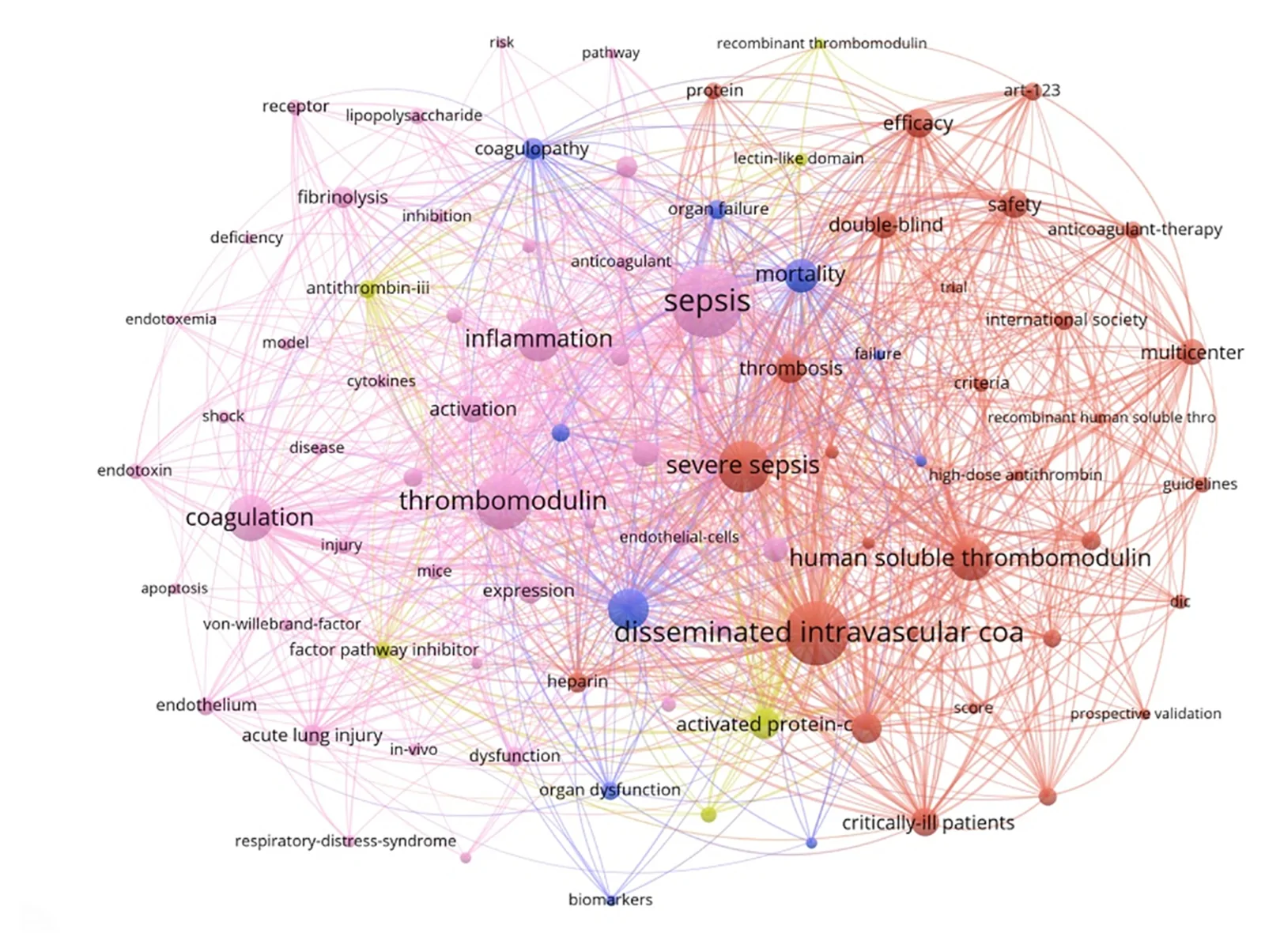
Co-occurrence network of high-frequency keywords (≥13 occurrences) in TAT/TM-related SIC diagnosis research. SIC = sepsis-induced coagulopathy, TAT = thrombin-antithrombin complex, TM = thrombomodulin.

**Figure 12.**
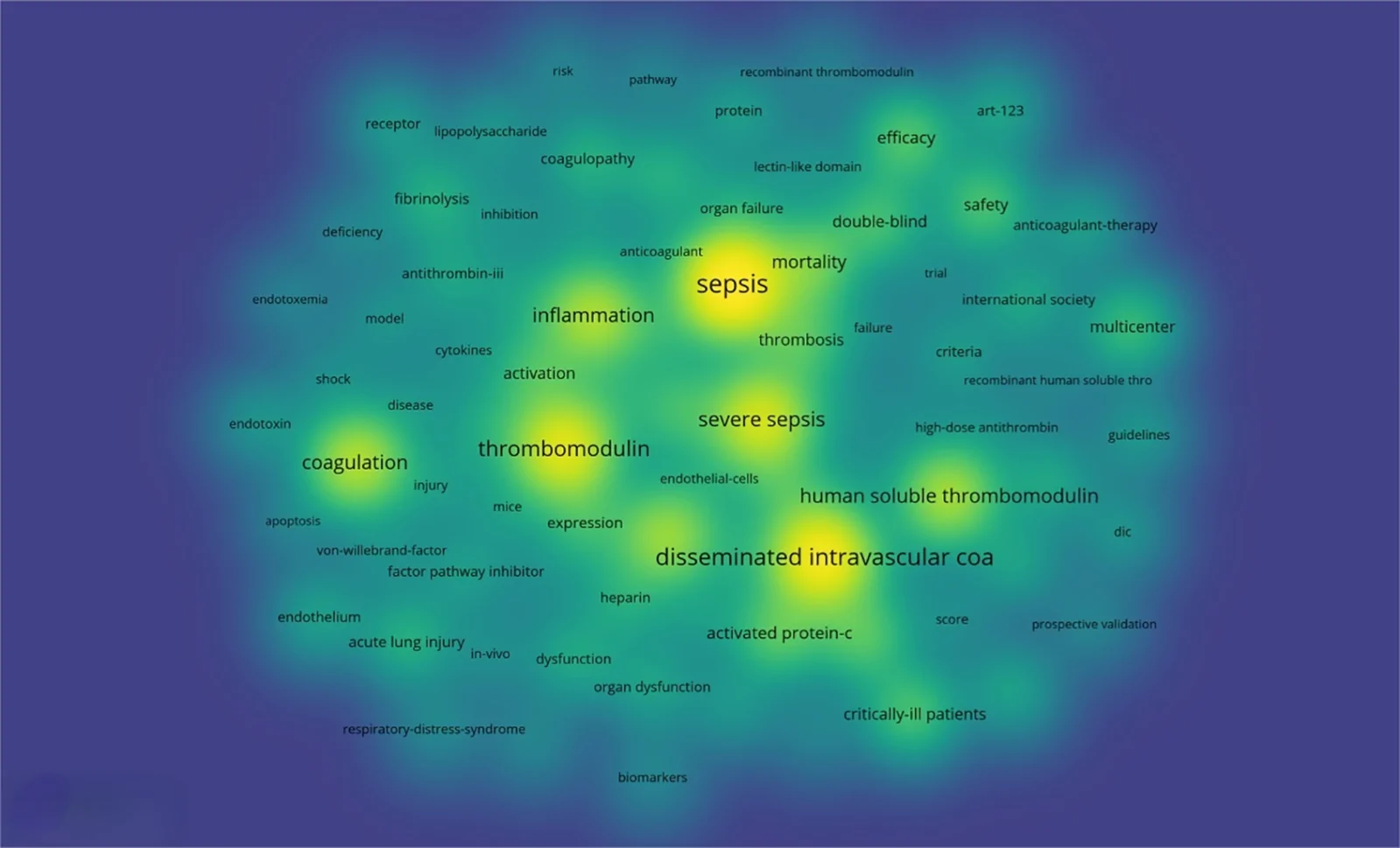
Density map of high-frequency keywords in TAT/TM-related SIC diagnosis research. SIC = sepsis-induced coagulopathy, TAT = thrombin-antithrombin complex, TM = thrombomodulin.

**Figure 13.**
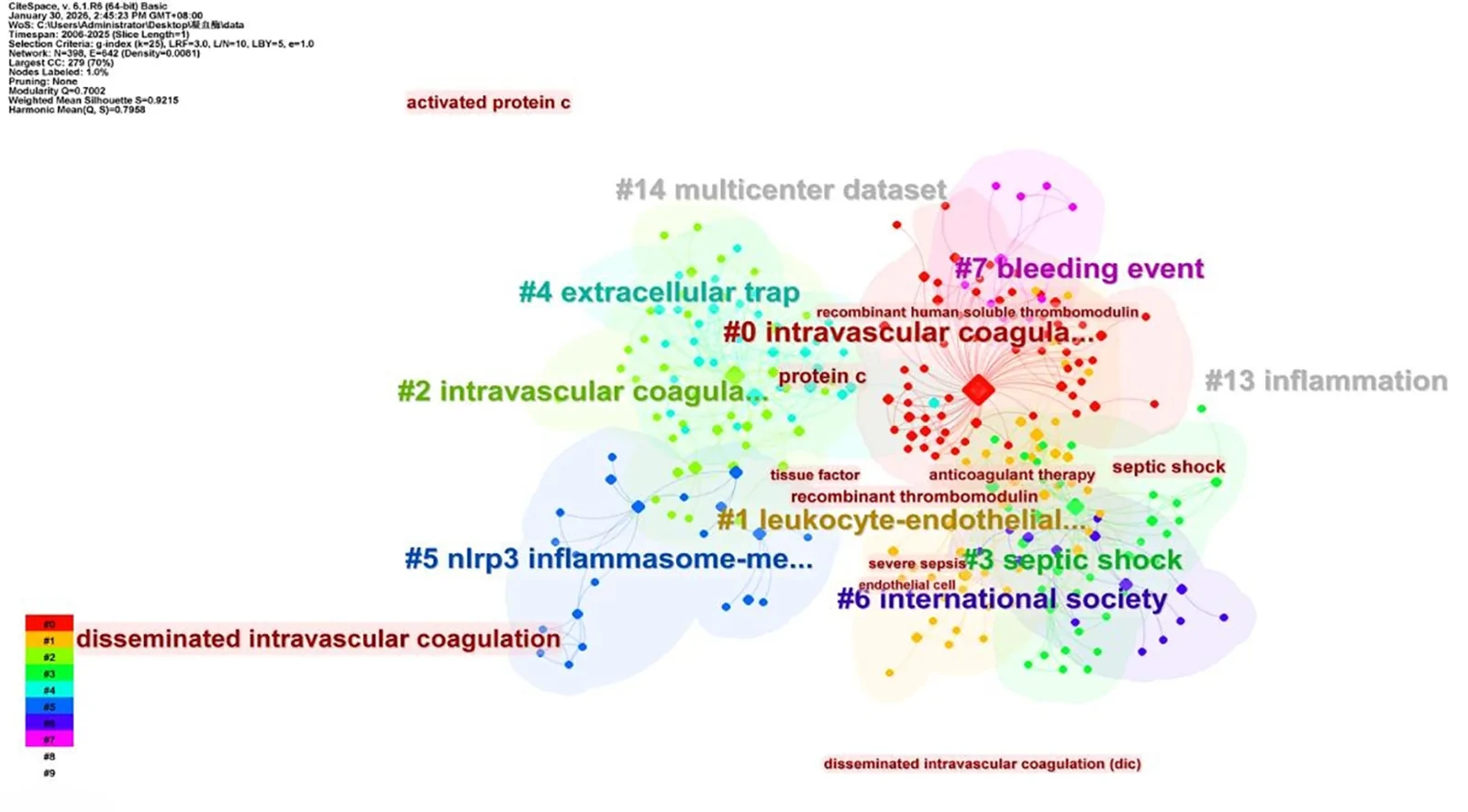
Keyword clustering diagram illustrating core research directions in TAT/TM-related SIC diagnosis. SIC = sepsis-induced coagulopathy, TAT = thrombin-antithrombin complex, TM = thrombomodulin.

Node size represents keyword frequency; node color indicates different thematic clusters. Thicker lines signify stronger co-occurrence relationships.

The color gradient indicates local keyword density: warmer colors (yellow/red) represent higher density (greater research concentration), while cooler colors (blue/green) denote lower density. Keyword label sizes reflect occurrence frequency.

Node size corresponds to keyword frequency; node colors differentiate thematic clusters (e.g., clusters #1–#14), visually representing the thematic structure and primary research directions.

### 3.9. Keyword burst analysis

The top 25 burst keywords (Fig. 14) identified key emerging frontiers from 2006 to 2025, revealing dynamic shifts in research hotspots and frontiers over time. The most recent burst keywords (2023–2025) include “SIC,” “endothelial cell,” and “anticoagulant therapy for SIC in ICUs,” representing current and future core research directions in this field.

**Figure 14.**
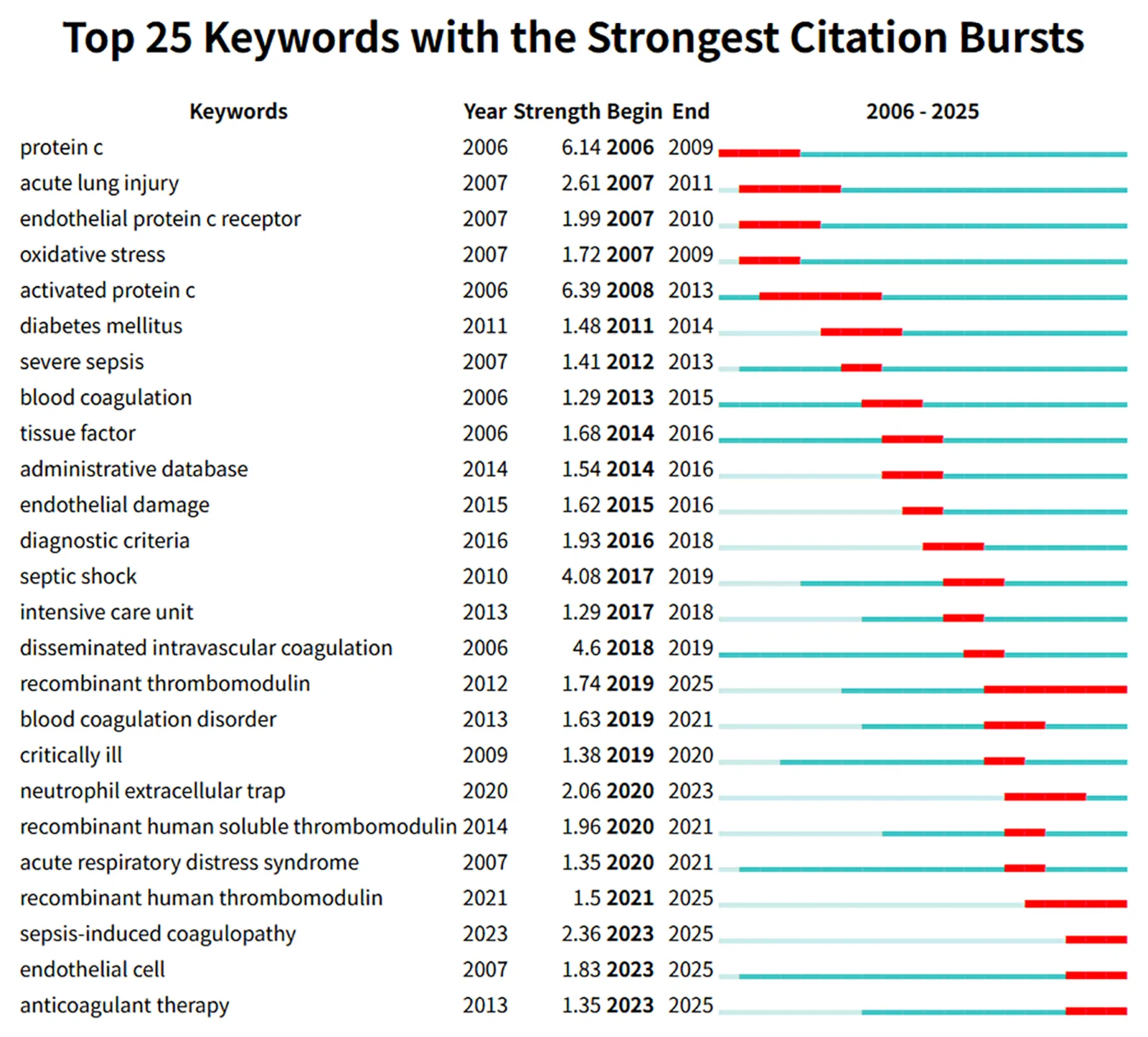
Burst keyword analysis of TAT/TM-related SIC diagnosis research (2006–2025). SIC = sepsis-induced coagulopathy, TAT = thrombin-antithrombin complex, TM = thrombomodulin.

The burst of “endothelial cell” indicates increased attention to endothelial cell injury, which is a fundamental pathological feature of SIC. The burst of “anticoagulant therapy for SIC in ICUs” reflects the urgent clinical demand for optimized anticoagulant treatment strategies. Additionally, the emergence of “SIC” emphasizes precise SIC diagnosis as a significant research priority, particularly through the combined detection of multiple biomarkers to overcome limitations inherent in single-biomarker approaches (TAT and TM alone). Figure 14 identifies “anticoagulant therapy” as a long-term burst keyword, underscoring its persistent research importance. Figure 15 highlights recent influential literature by Vincent JL and Levi M, providing critical insights supporting current research frontiers.

**Figure 15.**
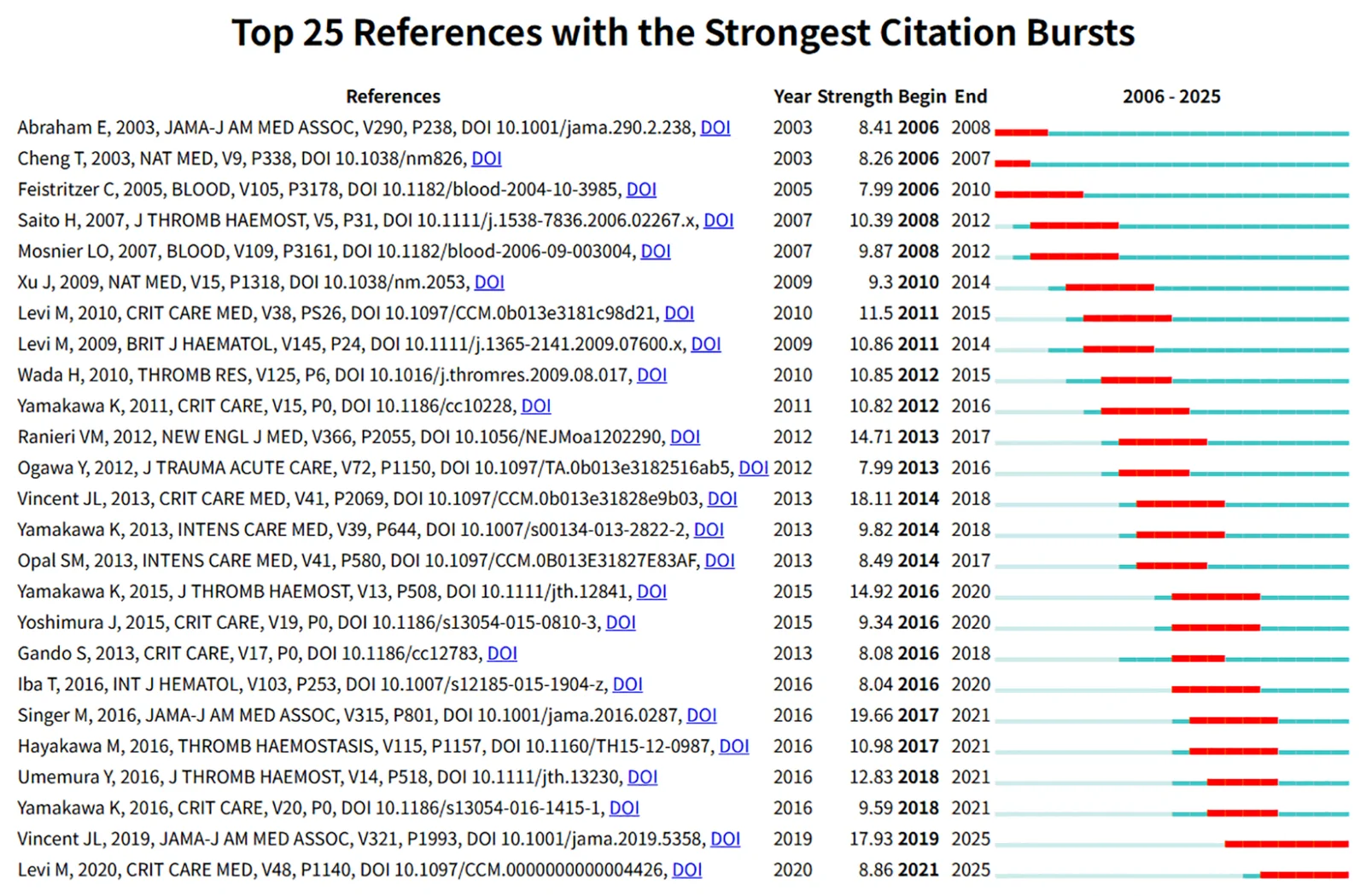
SIC = sepsis-induced coagulopathy, TAT = thrombin-antithrombin complex, TM = thrombomodulin.

## 4. Discussion

### 4.1. Overall development trends

Our results indicate that publications concerning TAT and TM in SIC diagnosis from 2006 to 2025 followed an “increase-peak-decline” pattern, closely associated with updates to SIC diagnostic criteria, advancements in detection technologies, and major public health events. Publication growth during 2006 to 2015 was primarily driven by evolving definitions of sepsis (especially the Sepsis-3.0 criteria)^[13,14]^ and increased application of TAT and TM assays, collectively fostering substantial progress in basic and clinical research. The publication peak between 2016 and 2018 was notably influenced by the 2016 International Consensus on Sepsis-3.0 and the establishment of the first diagnostic criteria for SIC in 2017,^[5]^ which incorporated TAT measurement and stimulated extensive global confirmatory studies. Since 2019, however, publication numbers have steadily declined, suggesting the field has reached a period of stagnation, characterized by challenges in deep integration and innovation. This stagnation is largely attributable to a lack of personalized diagnostic strategies for critically ill patients with varied disease severities.

Key factors include: increasing recognition of the limitations of individual TAT and TM assessments, such as insufficient specificity and real-time diagnostic capabilities^[7,8]^; and the impact of the COVID-19 pandemic beginning in 2020, which redirected substantial global research resources towards COVID-19-related coagulation disorders, consequently limiting progress in TAT/TM-focused SIC diagnosis research.^[14]^ Notably, a recent bibliometric analysis by Zhang et al (2024) broadly examined SIC research trends but did not specifically investigate the roles of TAT and TM as combined biomarkers.^[15]^ In contrast, our study represents the first comprehensive 20-year bibliometric review specifically focusing on TAT and TM in SIC diagnosis, covering critical periods of diagnostic criteria updates. Thus, our findings comprehensively capture the developmental trajectory of this specific research area, filling a gap in targeted analyses of these core biomarkers.

### 4.2. International research landscape

Japan, the United States, and China ranked as the top 3 countries in publication output, reflecting significant investment and research activities. However, notable differences exist regarding their international influence and recognition. The United States leads in citation impact and serves as a central collaborator despite fewer publications than Japan, highlighting its academic prominence and robust international collaboration with countries such as Japan, China, and the Netherlands.^[6,9]^ Japan, though first in publication quantity, has a relatively low average citation frequency, suggesting room for increased international recognition. Japan’s research has primarily involved domestic collaborations, with relatively limited international partnerships. China ranks third in publication quantity but surpasses Japan in average citation rate (42.57 citations per article), indicating substantial international recognition. Nevertheless, compared with developed countries such as the United States and the United Kingdom, China has significant potential to increase publication volume and academic influence.^[10,16]^ Given its secondary role in international collaborations, China should further strengthen high-level collaborations with leading research teams from the United States (e.g., University of Pittsburgh, National Institutes of Health) and European institutions (e.g., University of Amsterdam, Juntendo University), leveraging complementary expertise in biomarker validation, clinical trial design, and translational medicine to enhance academic visibility. Institutional analysis revealed stable core research teams supported by strong medical research infrastructures, acting as the primary driving force in this field. However, institutions worldwide mainly focus on domestic collaborations, with limited high-level international cooperation,^[17]^ potentially restricting innovation and development. In the future, overcoming geographic barriers through enhanced international exchanges and cooperation among institutions is crucial for promoting knowledge dissemination and translational application of research findings.

### 4.3. Analysis of core issues and emerging trends

Keyword co-occurrence and clustering analyses indicate that research hotspots primarily encompass the fundamental pathological mechanisms underlying sepsis and SIC, the diagnostic utility of TAT and TM as SIC biomarkers, interactions between sepsis-associated coagulation disorders and inflammatory responses, and anticoagulant therapeutic strategies for SIC.^[18]^ The evaluation of TAT and TM as early SIC biomarkers represents a central theme, reflecting the critical pathological processes involving coagulation activation and endothelial cell injury. Keyword burst analysis identified SIC, endothelial cell injury, and anticoagulant therapy for SIC in ICUs as forefront topics between 2023 and 2025, highlighting current and emerging research directions.

Consistent with the 2024 SIC diagnostic guidelines updated by the International Society on Thrombosis and Haemostasis, endothelial cell injury has been recognized as a pivotal factor in SIC pathogenesis.^[19]^ Recent studies have emphasized its therapeutic potential by modulating endothelial barrier functions and mitigating coagulation activation.^[15,20]^ As a specific biomarker of endothelial damage, TM correlates closely with SIC severity and patient prognosis, emerging as a significant research focus. Additionally, refining anticoagulant therapeutic strategies for critically ill patients with varying degrees of coagulation disorders has become increasingly important. This includes the development of novel anticoagulant agents (e.g., recombinant soluble TM) and exploration of combined treatment modalities.^[7,11]^ Precise SIC diagnosis, such as constructing diagnostic models integrating multiple biomarkers, represents another core research direction, aimed at addressing the limitations associated with single-biomarker approaches.^[4,8]^

For instance, recent clinical studies demonstrated promising diagnostic performance when combining TAT and TM with neutrophil extracellular traps (NETs) or tissue factor (TF),^[8,15]^ aligning with the emerging trend of multi-biomarker combination approaches identified in the keyword analysis.

### 4.4. Study limitations

Several inherent limitations of this study should be acknowledged. First, literature retrieval was restricted to PubMed and WoSCC, excluding other significant databases such as CNKI and Embase. This may have led to the omission of relevant publications, particularly important European and Chinese literature. Future studies could include these databases to enable cross-regional validation and enhance sample comprehensiveness. Second, the search language was limited to English, excluding non-English literature (e.g., Chinese publications), potentially biasing the analysis regarding research output from non-English-speaking countries, such as China. Third, reliance exclusively on bibliometric data confines the analysis to quantitative metrics, unable to directly evaluate the substantive quality of individual studies. Fourth, VOSviewer software does not support direct integration of data from multiple databases; therefore, the collaborative network analysis in this study was based solely on PubMed data, possibly limiting insights into the full scope of international cooperation. Future research might employ advanced visualization tools (e.g., Gephi) capable of integrating multi-source data for more comprehensive network analysis.

### 4.5. Prospects for future research directions

To further advance this field, future studies guided by our findings and current research trends should prioritize the following objectives. First, develop a multi-biomarker detection system tailored for critically ill patients in ICUs by combining TAT/TM with novel biomarkers (e.g., NETs, tissue factor).^[6,8]^ Such systems would facilitate early and accurate SIC diagnosis, enhancing both diagnostic specificity and sensitivity. Second, conduct comprehensive research on the pathological mechanisms of SIC, particularly focusing on interactions among coagulation pathways, inflammatory responses, and endothelial cell function, to identify novel therapeutic targets.^[18]^ Third, refine anticoagulant therapeutic protocols for SIC by initiating large-scale, multicenter clinical trials to confirm the efficacy and safety of emerging anticoagulants (e.g., recombinant soluble TM),^[7,11]^ and exploring personalized anticoagulant regimens. Fourth, enhance international academic collaboration by overcoming geographical barriers among countries and institutions, promoting large-scale cooperative studies, and increasing the international recognition and clinical translational potential of research findings.

## 5. Conclusion

From 2006 to 2025, research on TAT and TM in SIC diagnosis established a global landscape prominently involving Japan, the United States, and China. The United States occupies a leading position in terms of academic influence and international collaboration, although high-level cross-country and cross-institutional cooperation remains relatively limited. Research hotspots include the diagnostic application of TAT and TM, the pathogenesis of SIC, and anticoagulant therapies for SIC in ICUs. Precise SIC diagnosis, endothelial cell injury, and optimized anticoagulant treatment strategies represent critical current and future research frontiers. Currently, the field faces challenges including limitations associated with single-biomarker diagnostics and fragmented global research efforts.

Future directions should emphasize strengthening international academic collaboration, particularly by developing multi-biomarker diagnostic models (e.g., integrating TAT/TM with NETs), incorporating multi-omics methodologies, and exploring novel anticoagulant therapies. These endeavors will establish a solid theoretical framework for the early and precise diagnosis and personalized management of SIC in critically ill patients, thereby providing valuable clinical guidance in critical care medicine.

## Acknowledgments

The author gratefully acknowledges the valuable guidance on literature and dosage from the supervisor. Sincere thanks are also extended to colleagues for their support. Finally, the author would like to thank the anonymous reviewers and editors for their constructive comments and suggestions on this manuscript.

## Author contributions

**Conceptualization:** Yidan Zhao.

**Data curation:** Yidan Zhao, Huaxing Yao.

**Formal analysis:** Yidan Zhao, Huaxing Yao.

**Investigation:** Huaxing Yao, Pingping Xiao, Amei Li.

**Methodology:** Yidan Zhao, Huaxing Yao, Dong Lai.

**Project administration:** Yidan Zhao.

**Writing – original draft:** Yidan Zhao.

**Writing – review & editing:** Yidan Zhao, Huaxing Yao, Dong Lai, Pingping Xiao, Amei Li.


